# Observational Case Analysis of Neonates With Large Cephalohematoma

**DOI:** 10.7759/cureus.14415

**Published:** 2021-04-11

**Authors:** Melih Üçer, Abdullah E Taçyıldız, Ilhan Aydın, Nesrin Akkoyun Kayran, Semra Işık

**Affiliations:** 1 Neurosurgery, University of Health Science Kanuni Sultan Süleyman Research and Training Hospital, Istanbul, TUR; 2 Neurosurgery, School of Medicine, Karabuk University, Karabük, TUR

**Keywords:** cephalohematoma, management, ossification

## Abstract

Introduction

Cephalohematomas in the newborn period are related to the accumulation of blood between the bone and periosteum as a result of a series of adverse conditions during labor. The optimal approach to cephalohematoma cases is still unclear. In this study, we aimed to present the follow-up data of 94 newborns with a cephalohematoma size of >50 mm and a higher risk of ossification.

Methods

This is a single-center, non-randomized, prospective, observational study conducted from May 2014 to May 2019. Records of all newborns with cephalohematoma were reviewed in terms of gender, birth weight, cephalohematoma region, transverse/vertical diameter of the lesion, delivery method, and rate of ossification.

Results

The girl-to-boy ratio was 53/41, with a mean gestational age of 38.3±1.4 weeks and a mean birth weight of 3,300±800 grams. The mean transverse/vertical diameter of cephalohematoma was 59±9 mm. Cephalohematoma was completely resorbed at the first-month control visits in 72 (76.6%) cases, whereas nine (9.57%) had an ossified cephalohematoma. The ossification was completely or partially resorbed in these at the end of the one-year follow-up.

Conclusion

Hence, we suggest that an early intervention is not required in the routine treatment of cases with hematomas with a size of >50 mm in size unless otherwise stipulated with clinical indications.

## Introduction

Birth traumas include a wide spectrum of incidents that can occur from the initiation and termination of the labor, which might have structural and/or functional effects on the newborn. The result of these traumas ranges from paralysis to cephalohematoma. Infant cephalohematoma is a subperiosteal blood accumulation observed in 0.2%-3% of newborns, usually localized over the parietal region, and markedly delimited by suture lines [1,2].

Since the coagulated blood is slowly absorbed, the resorption of cephalohematoma takes several weeks, while most newborns recover within the first four weeks of the neonatal period. Delayed resorption may often lead to progressive calcification and ossification [2]. Ossified cephalohematomas are cosmetic asymmetries and deformities of the skull and mostly heal spontaneously via the gradual absorption of the ossified tissue by the expanding calvarium, and the physical appearance usually turns to normal before the age of one year [3].

On rare occasions, surgical intervention is required for the treatment of persistent ossified cephalohematoma cases in order to prevent a progression to undesirable sequelae during the developmental duration.

The aim of this prospective study was to evaluate the management outcomes of 94 newborns who were admitted to the neurosurgery clinic of our hospital due to a cephalohematoma, with a transverse/vertical diameter of >50 mm, and a higher risk of ossification.

## Materials and methods

This is a single-center, non-randomized, prospective, observational study conducted at the Neurosurgery Clinic of Kanuni Sultan Süleyman Research and Training Hospital, Istanbul, Turkey, between May 2014 and May 2019. The study was conducted in accordance with the Declaration of Helsinki following approval of the Local Ethical Committee. Informed consent obtained was obtained from the parents or legal guardians of all patients.

Patients were evaluated in terms of gender, birth weight, delivery method, cephalohematoma region, transverse/vertical diameter of the hematoma, and rate of ossification.

Infants with an abnormal coagulation profile (suspected or confirmed hemophilia, thrombocytopenia, disseminated intravascular coagulation) and central nervous system anomalies were excluded from the study.

At admission to the neurosurgery outpatient clinic, patients were applied compressive dressing and called for a check-up at the first month, second month, and first year.

Computed tomography (CT) scan is indicated only when neurological symptoms develop, when there is a possibility of a collapsed skull fracture, or when a morphologic deformity of the skull is considered. None of the patients underwent CT scan, except for patients whose cephalohematoma did not regress by the end of the second postnatal month.

## Results

A total of 94 neonates with cephalohematoma were referred to our outpatient clinic, of whom 53 (56.4%) were girls and 41 (43.6%) were boys, with a mean gestational age of 38.3±1.4 weeks and mean birth weight of 3,300±800 grams. Of the patients, 69 (73.4%) were born via vaginal delivery and 25 (26.6%) were delivered by cesarean section. The mean transverse/vertical diameter of cephalohematoma measured by ultrasonography (USG) in the first examination was 59±9 mm. Cephalohematoma was detected at the parietal region in 71 (75.5%) infants, at the occipital region in 12 infants (12.8%), and at the temporoparietal region in 11 infants (11.7%).

**Figure 1:**
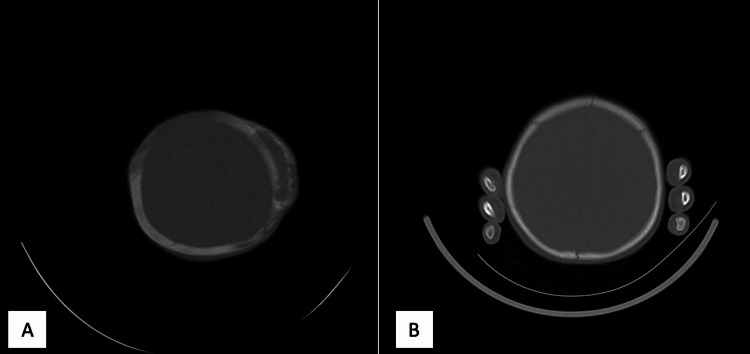
(A) An ossified cephalohematoma was seen in the left parietal region in a two-month-old baby. (B) Ossification was completely resorbed in the control CT taken at the ninth month.

**Figure 2:**
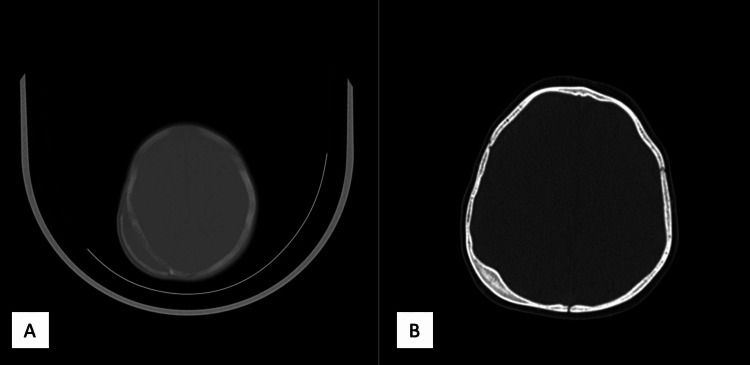
(A) An ossified cephalohematoma was seen in the right parietal region in a two-month-old baby. (B) At the 12th month, the ossified hematoma is seen to be resorbed in the control CT scan, but cranium thickening is observed in the area where the cephalohematoma is present.

After compressive dressing, cephalohematoma was completely resolved in 72 (76.6%) cases at the first-month control visits and in 12 (12.7%) cases at the second month of follow-up. Of the remaining 10 newborns who were followed up with intermittent controls, ossification formation in cephalohematomas was observed in nine (9.57%) patients. At the end of the one-year follow-up, the ossification was completely or partially resorbed in all nine patients (Figures 1, 2). None of the patients had additional clinical findings or adverse events related to the condition, and surgery was not required for any patients.

## Discussion

**Figure 3:**
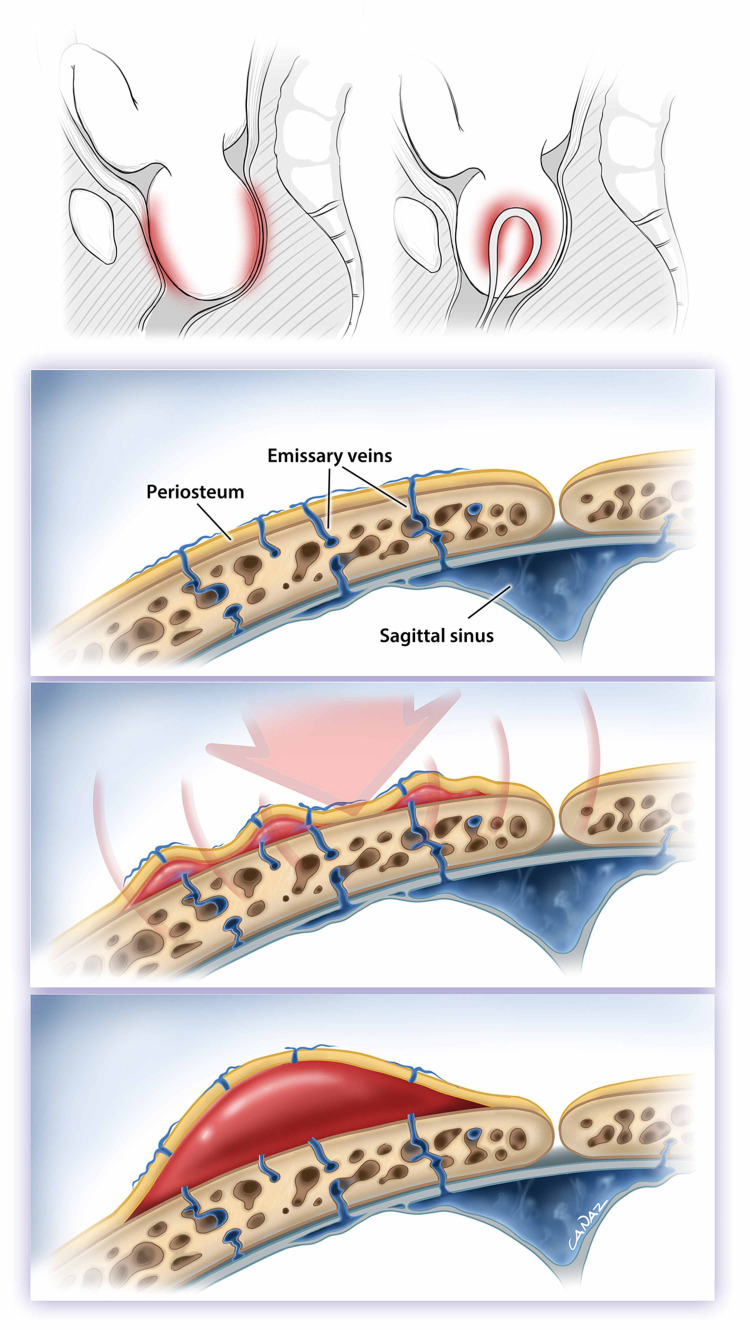
Cephalohematoma mechanism. Printed with permission from Dr. Gokhan CANAZ©, Department of Neurosurgery, Arnavutkoy State Hospital.

The exact mechanism of development of cephalohematoma during labor is unknown; however, it is thought to be an injury caused by forceful and repeated compression of the skull by the pelvic bones alongside contractions during labor. This shear action leads to bleeding of the diploic vessels to the subperiosteal layer of the skull [4] (Figure 3). Common risk factors are prolonged labor, macrosomia, weak and ineffective uterine contractions, male gender, abnormal presentation and position of the fetus, forceps- or vacuum-assisted delivery, polycyesia, and cephalopelvic disproportion [5]. It has been reported that the risk of cephalohematoma development increases by up to 10.8% with assisted vaginal delivery [6]. Cephalohematomas are mostly located on the parietal bone and on the right side, but they are bilateral in 10% of the cases [7].

Despite the fact that for unknown reasons the incidence of cephalohematoma is reported to be twice as higher in males than females in the literature, in our series the majority of the cases were female [8]. The overall incidence of cephalohematoma was approximately 1.25% per year for our center, which is one of the largest hospitals in a European metropole and specializes in women’s and children’s health with a birth incidence of more than 5,000 cases per year [9].

Although the tissue formed when cephalohematoma is not resorbed was previously considered to be calcification, studies have shown that the phenomenon is ossification [10]. It is essential to be thoroughly familiar with the embryology of the scalp in order to better understand the ossification process of cephalohematoma. During the developmental process, the scalp begins to appear as a vascular plexus on the 49th day of gestation. A few weeks after the first hair begins to appear on the eyebrows at the 16th week, they begin to cover the scalp, and the fetal hair, which is longer than lanugo, begins to grow at the seventh month of the intrauterine period. The structure of the scalp is different from the skin structures of other body parts owing to the inductive interaction between the two different germ layers, ectoderm and mesoderm [11]. On the other hand, the periosteum, which is very effective in the growth, repair, and blood supply of the bone, is not yet fully understood and has been a subject of still existing controversy and debate [12].

The periosteum histologically consists of outer and inner layers. The superficial part of the outer "fibrous layer” contains a higher level of vascularization with a rich neural network and significantly contributes to the blood flow of the bone and adjacent muscle tissue. The profound section of the outer layer has less vascularization and is collagenous with a small number of cells. On the other hand, the inner "cambium layer" is rich in cells, consisting of mesenchymal progenitor cells, differentiated osteogenic progenitors, osteoblasts, and fibroblasts. Osteoblasts are in contact with the bone layer, and pericytes with high osteoblastic potential are also found in the region due to rich vascular network [12]. The cambium layer is thickest in fetal life and gets thinner with age [13]. The calvarial periosteum, which is the area of interest in our study, has less osteogenic potential compared to the other forms of periosteum [14,15].

A reciprocal interplay between osteogenic progenitor cells of the periosteum, cytokines, and growth factors in the hematoma along with birth trauma have been found to play a role in the formation of cephalohematoma ossification [16]. Cephalohematoma progresses to ossification in approximately 3%-5% of cases [17,18]. Although there is a controversy between the initial size of cephalohematoma and its progression to ossification in the literature, data from case reports suggest that massive cephalohematomas at presentation have a higher potential for progression to ossifying cephalohematomas [19].

In our study, the rate of ossification was 9.57%, and we believe that the relatively higher ratio in our study might be due to a selection bias since we included only the cases with a transverse/vertical diameter of >50 mm. No ossification was observed in any newborns with a cephalohematoma transverse/vertical diameter below 50 mm, which we did not include in the study (data not shown).

**Figure 4:**
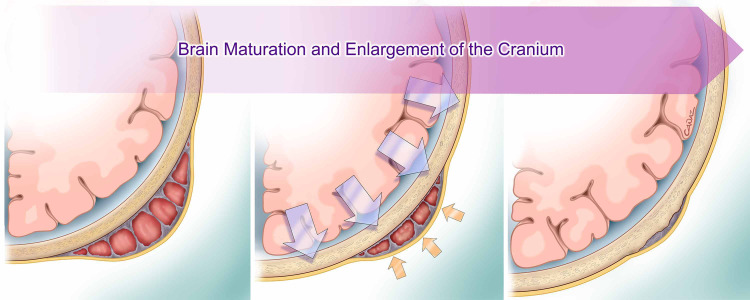
The ossified tissue by the expanding calvarium, and the physical appearance usually turns to normal before the age of one year. Printed with permission from Dr. Gokhan CANAZ©, Department of Neurosurgery, Arnavutkoy State Hospital.

The optimum treatment of choice for cephalohematoma is still controversial due to its potential for a benign clinical course. Hence, the common approach is usually observational and conservative as the cephalohematoma is gradually absorbed as the skull develops; however, this process usually takes three to six months [20] (Figure 4).

In some reports, aspiration is recommended for nonabsorbable cephalohematoma after one month, whereas some do not advise aspiration due to the increased risk of infection [21-23].

Firlik and Adelson suggested puncture for all cephalohematoma cases after one month of observation in order to avoid scalp deformity and delayed open surgery [24]. Another study advocated aspiration under local anesthesia earlier than one month to avoid difficult evacuation as a result of premature ossification [25]. The procedure was performed using a 20G spinal needle, without a drain insertion, and a routine coverage with sterile surgical dressing for 24 hours. Eseonu et al. reported an early surgical evacuation of a large cephalohematoma (>70 mm) by small scalp incision under general anesthesia in a neonate at two weeks of age, suggesting that a complicated surgery might be avoided by early intervention [26].

Despite their promising data with the surgical aspiration of both large and unossified cephalohematoma cases, both methods have some potential restraints. Firstly, although local anesthetic drugs of the amide group are usually considered safe, they may cause adverse events and toxic effects in infants even in case of an administration of the minimal dose. The reports have suggested toxic activity and methemoglobinemia in various surgical practices of the infant with these agents [27]. It should be noted that systemic interference might be possible with the absorption of the anesthetic agent when applied on the areas with higher absorption rate, and minimal portions of local anesthetic agent might also penetrate through the ruptured small blood vessels in the cephalohematoma cases into the systemic circulation. Also, the possible causes of impaired clearance and G6PD deficiency should be of concern while local anesthetics are given to neonates, and the procedures might be performed in operating room conditions instead of an outpatient setting in order to provide access to emergency intervention condition in case of toxicity.

On the other hand, surgery of infants possesses its own specific drawbacks, including the requirement of fasting, insertion of a drain for larger hematoma cases, and limited access of the mother and caregiving medical team to the newborn to nurse and calm down the newborn during the postoperative follow-up period. In addition, needle aspiration is related to an increased incidence of infection mainly with strains of *Escherichia coli* and *Staphylococcus aureus*; however, cephalohematoma itself is a risk factor for spontaneous infection with these agents [28]. Hence, close observation of the infant is essential following an invasive procedure, in the presence of a large hematoma, or during any incidents of irritability, fever, or lethargy in parallel to laboratory examination with markers of local or systemic infection. An USG-guidance and use of an echogenic needle are recommended for the needle aspiration process to rule of puncture-related complications.

Despite the diversity and lack of definite consensus on treatment opinions, the most widely accepted criteria for surgery includes cases with cosmetic deformity, confirmed cases of restricted brain growth, and cases with associated craniosynostosis [2,29]. Surgery might be challenging since complicated cases with ossification undergo surgical reshaping of the skull via trimming the deformed bony prominences, and experience is needed to reconstruct the depressed skull portions in order to correct the cosmetic deformity and restore the anatomical form [30].

Although the ossification rate was 9.57% in our series, we did not apply any kind of surgical intervention and observed that all ossifications of the developing cases are resorbed within one year; thus, we recommend a close observation with concomitant follow-up visits until the age of one year prior to an ultimate surgical decision.

## Conclusions

Cephalohematoma is usually a lesion that disappears spontaneously within the first few months of postnatal life without any cosmetic deformities. Ossifying cephalohematoma is a rare clinical condition. Since any intervention is generally not required in the routine treatment of cases with hematomas > 50 mm in size, unless there is a clinical indication, surgery should be performed only for cosmetic purposes after the age of one year.
